# 

**Published:** 1897-10

**Authors:** 


					﻿ESTABLISHED >*55.

ATLANTA
MEDICO AND SU WGAIl
JOURNAL.
newt’seiue8.	Atlanta, Ga., October, 1897. No. 8.\
				

